# Does Reinfusion of Stem Cell Products on Multiple Days Affect Engraftment?

**DOI:** 10.4274/tjh.2018.0071

**Published:** 2018-11-13

**Authors:** Şerife Solmaz Medeni, Doğuş Türkyılmaz, Celal Acar, Ömür Gökmen Sevindik, Faize Yüksel, Özden Pişkin, Mehmet Ali Özcan, Fatih Demirkan, Bülent Ündar, İnci Alacacıoğlu, Güner Hayri Özsan

**Affiliations:** 1 University of Health Sciences, İzmir Bozyaka Training and Research Hospital, Clinic of Hematology, İzmir, Turkey; 2 Dokuz Eylül University Faculty of Medicine, Department of Hematology, İzmir, Turkey; 3 Fırat University Faculty of Medicine, Department of Hematology, Elazığ, Turkey

**Keywords:** Multiple myeloma, Autologous transplantation, Multiple reinfusion days

## Abstract

**Objective:**

High-doses of melphalan treatment with autologous stem cell transplantation in multiple myeloma (MM) remains a major treatment modality in suitable patients. A minimal dose of 2x10^6^/kg CD34+ cells is preferred to achieve engraftment. Some patients need multiple leukapheresis procedures to achieve the necessary number of CD34+ cells, but this can cause a high volume of stem cell product that cannot be given in a single day. Whether or not the number of infusion days affects engraftment has not been studied before. We aimed to evaluate the impact of reinfusion of stem cells on multiple days on engraftment results.

**Materials and Methods:**

Demographic features, CD34+ cell doses, neutrophil and platelet engraftment days, hospitalization days, and number of infusion days of 149 autologous transplantations of 143 MM patients were evaluated retrospectively.

**Results:**

The data of 143 MM patients who were transplanted were analyzed retrospectively. Median age was 55±8.5 (range: 26-70) years with a male/female ratio of 91/58. Hospitalization days for all patients were 24±6 (range: 14-50) days. Mean CD34+ cell number was (7.5±5.3)x10^6^/kg (range: 1.5-31x10^6^/kg). CD34+ cells were reinfused in 1 day in 80.5% (n=120) of the patients, 2 days in 18.2% of the patients (n=27), and 3 days in 1.3% of the patients (n=2). For 29 patients, reinfusion was applied in more than 1 day because of the high volume of stem cell product. We did not see any dimethyl sulfoxide toxicity, cardiac arrhythmia, or volume overload complications. Hypertensive attacks during infusion were easily controlled by furosemide treatment. In the group with multiple infusions, the infused CD34+ cell numbers had a mean of (4.8±2.8)x10^6^/kg, and in the single infusion group the mean was (8.1±5.5)x10^6^/kg. There were no statistical differences between the two groups regarding platelet and neutrophil engraftment days (p=0.850, r=0.820 and p=0.500, r=0.440). There was no statistical difference between the two groups for hospitalization days (p=0.060, r=0.050).

**Conclusion:**

In cases with a high volume of stem cell product to acquire adequate stem cells, reinfusion can be safely applied across multiple days without any delay in engraftment.

## Abstract (tr)

**Amaç:**

Multipl myelom (MM) hastalarında yüksek doz melfalan ve otolog kök hücre nakli fit hastalarda hala majör tedavi modalitesidir. Yeterli engrafman için en az 2x10^6^/kg CD34+ hücre dozu tercih edilir. Bazı hastalar gerekli sayıda CD34+ hücre sayısına ulaşmak için çoklu lökoferez prosedürüne ihtiyaç duyarlar, fakat bu tek günde verilemeyen yüksek miktarda kök hücre ürününe neden olabilir. İnfüzyon günü sayısının engrafmanı etkileyip etkilemediği daha önce çalışılmamıştır. Çoklu gün kök hücre reinfüzyonunun engrafman sonuçlarına etkisini değerlendirmeyi amaçladık.

**Gereç ve Yöntemler:**

Demografik özellikler, CD34+ hücre dozları, nötrofil ve trombosit engrafman günleri, hastanede yatış günleri ile 143 MM hastasının 149 otolog transplantasyonunun infüzyon gün sayısı retrospektif olarak değerlendirildi.

**Bulgular:**

Transplantasyon yapılan 143 MM hastasının verileri retrospektif olarak incelendi. Ortalama yaş 55±8,5 idi (en düşük: 26 - en fazla: 70) ve erkek/kadın oranı 91/58 idi. Tüm hastalar için hastanede kalış günleri 24±6 gündü (en düşük: 14 - en fazla: 50). Ortalama CD34+ hücre sayısı (7,5±5,3)x10^6^/kg idi (en düşük: 1,5 x10^6^/kg - en yüksek: 31x10^6^/kg). Hastaların %80,5’inde (n=120) tek günde CD34+ hücre reinfüzyon, hastaların %18,2’sinde (n=27) 2 günde CD34+ hücre reinfüzyon ve hastaların %1,3’ünde (n=2) 3 günde CD34+ hücre reinfüzyon yapılmıştı. Yirmi dokuz hastada yüksek hacimde kök hücre ürünü olması nedeniyle 1 günden fazla reinfüzyon uygulandı. Herhangi bir dimetil sülfoksit toksisitesi, kardiyak aritmi veya hacim yükü komplikasyonları görmedik. İnfüzyon sırasında hipertansif ataklar furosemid tedavisi ile kolayca kontrol altına alındı. CD34+ hücre sayıları ortalama çoklu gün reinfüzyonu yapılan grupta (4,8±2,8)x10^6^/kg ve tek gün reinfüzyon grubunda ise (8,1±5,5)x10^6^/kg idi. İki grup arasında trombosit ve nötrofil engrafman günleri açısından istatistiksel olarak anlamlı fark yoktu (p=0,850, r=0,820 ve p=0,500, r=0,440). İki grup arasında hastanede yatış günleri açısından istatistiksel fark yoktu (p=0,060, r=0,050).

**Sonuç:**

Yeterli kök hücre elde etmek için yüksek miktarda kök hücre toplanması durumunda, engrafmanda herhangi bir gecikme olmaksızın reinfüzyon çoklu günde güvenli bir şekilde uygulanabilir.

## Introduction

Multiple myeloma (MM) is a hematologic malignancy with a median age at presentation of 60-65 years [1]. High-dose melphalan therapy together with autologous stem cell transplantation (ASCT) in MM remains a major treatment modality in suitable patients [2]. Advanced age is a poor prognostic factor in studies using conventional chemotherapy even if the biological and clinical features in older MM patients are the same as to those of younger patients [3,4]. This procedure can be applied safely even in selected patients older than the age of 65. The use of peripheral stem cells was found to accelerate the speed of hematopoietic recovery, leading to a significant decrease in mortality and morbidity. ASCT has been a part of myeloma treatment in these patient groups [5,6,7]. Adequate collection and application of enough CD34+ hematopoietic stem cells are needed for successful transplantation. The determination of a suitable  minimum CD34+ cell dose for MM patients aged 65 years and older is critical given that the median age of diagnosis for MM is generally between 65 and 70 years of age and many of these patients may be transplant-eligible [8]. In recent practice, a CD34+ dose of at least 2x10^6^/kg is preferred for sufficient neutrophil and platelet engraftment, and ≥5x10^6^/kg CD34+ was shown to be related to a shortened time to platelet recovery [9,10].

The aim of our study was to examine the effect of CD34+ reinfusion on multiple days on engraftment results in MM patients. We evaluated the demographic features, infused CD34+ cell doses, neutrophil and platelet engraftment days, hospitalization days, and number of infusion days in 149 ASCTs of 143 MM patients retrospectively.

## Materials and Methods

Between February 2009 and September 2017, 143 patients with MM underwent ASCT at the Dokuz Eylül University Hospital Division of Hematology. Data were collected from the electronic and patient files of the medical archives retrospectively. Baseline patient characteristics are shown in Table 1. All patients were suitable for ASCT therapy and had enough stem cell collection. However, second ASCTs were planned for six patients due to late relapse of the disease. All patients were informed about the benefits and risks related to stem cell collection and transplantation. The majority of the patients had one or two lines of prior chemotherapy (range: 1-3) in the pre-ASCT period (vincristine, adriablastin, and dexamethasone [VAD] therapy; bortezomib and dexamethasone therapy; or lenalidomide and dexamethasone therapy).

Transplant details including mobilizing agents, CD34+ cell doses, neutrophil and platelet engraftment days, hospitalization days, and number of CD34+ reinfusion days were analyzed. The data were examined according to the number of CD34+ cell reinfusion days. The neutrophil and platelet engraftment days, hospitalization days, and collected CD34+ cell count of the patients for whom CD34+ cells had been reinfused in one day were compared to those of the patients for whom CD34+ cells been reinfused on multiple days. At the time of transplant, only 11.4% (n=17) of patients had complete response (CR). The majority (n=86, 57.6%) had achieved a very good partial response (PR), while 28% (n=42) of the patients had reached PR and 3% (n=4) had refractory or progressive disease. Second autologous transplantations were planned for six patients because of progressive disease.

Peripheral blood stem cells were collected in 1-4 apheresis procedures (mean: 1.7), following mobilization regimens. We used intravenous cyclophosphamide at 2.4 g/m^2^ for one day with mesna and granulocyte colony-stimulating factor (G-CSF, 5 µg/kg/day subcutaneously) in 133 patients (89.3%), G-CSF alone in 7 patients (4.7%), and plerixafor plus G-CSF in 9 patients (6%) for mobilization. Apheresis was initiated when  the CD34+ cells of peripheral blood samples increased to >10 µL. Each sample was tested by flow cytometric analysis for the proportion of cells expressing CD34. The minimum goal CD34+ stem cell dose as a target for collection was >2x10^6^ CD34/kg for all autologous transplantations.

The regimen for conditioning consisted of melphalan for all patients. Melphalan was given at a dose of 200 mg/m^2^ for 124 patients (83.2%) and at a reduced dose of 140 mg/m^2^ for 25 patients (16.8%) due to reduced creatinine clearance (<50 mL/min). Patients received G-CSF once a day starting on day 1 after the infusion of stem cells until the time of engraftment.

### Statistical Analysis

Data were entered and analyzed using SPSS 21.0. Descriptive statistics were used for baseline characteristics, transplant-related factors, and posttransplant results. Differences in the distribution of variables between patient subsets were analyzed using the Pearson chi-square test/correlation test/t-test. All statistical analyses were performed at a critical significance level of  0.05, and p-values were reported.

## Results

We analyzed 149 autologous transplantations of 143 MM patients between February 2009 and September 2017 retrospectively. Mean age was 55±8.5 (range: 26-70) years with a 91/58 M/F ratio. There were no significant differences in platelet engraftment days, neutrophil engraftment days, reinfusion days, hospitalization days, or infused CD34+ dose with regards to sex distribution (Table 2).

Patients were separated into two age groups: those aged younger than 60 years and those older. There was no significant difference between the two groups in terms of platelet and neutrophil engraftment days, multiple day reinfusion rate, hospitalization days, or infused CD34+ cell dose (Table 3).

When we analyzed all patients, hospitalization days were 24±6 (range: 14-50). Mean CD34+ cell count was (7.5±5.3)x106/kg (range: 1.5-31x106/kg). Platelet engraftment days were 13.9±3 (range: 9-30) and neutrophil engraftment days were 11.5±1.5 (range: 8-17).

Higher reinfused CD34+ cell doses were associated with faster platelet and neutrophil engraftment (p=0.034 and p= 0.001). Hospitalization days decreased because of a better transplantation outcome with the higher reinfused CD34+ cell doses (p=0.001).

CD34+ cells were reinfused in one day in 80.5% of patients (n=120), 2 days in 18.2% of patients (n=27), and 3 days in 1.3% of patients (n=2). For 29 patients, reinfusion was performed on more than one day, because of the higher volume of stem cell product and according to the tolerability of the patients. However, reinfusion of peripheral blood mononuclear cells cryopreserved with dimethyl sulfoxide (DMSO) can be associated with toxic reactions. We know that infusion of product containing more than 1 g/kg of DMSO per day can lead to increased DMSO toxicity. As a result, the days of reinfusion were determined according to the performance status of our patients, the amount of product they had, and the amount of DMSO contained in the products. There were also statistical differences between the two groups in terms of mobilization days. Mobilization days were found higher in the multiple day infusion group than in the single day infusion group (2.2 days vs. 1.6 days, p=0.0001). We did not see any DMSO toxicity, cardiac arrhythmia, or volume overload complications. Hypertensive crisis  was easily controlled by diuretic treatment at the during infusion. CD34+ cell levels were a mean of (4.8±2.8)x10^6^/kg in the multiple day infusion group and (8.2±5.5)x10^6^/kg in the single day infusion group. The infused CD34+ cell count was found higher in the single day infusion group than in the multiple day infusion group (p=0.003). There were no statistical differences between the two groups in the case of platelet and neutrophil engraftment days (p=0.850, r=0.820 and p=0.500, r=0.420) or hospitalization days (p=0.060, r=0.050) (Table 4).

## Discussion

MM is a disease of the elderly. ASCT is an important treatment modality in symptomatic myeloma patients. Treatment options have expanded in the last decade with novel drugs, but ASCT still maintains its place in the treatment of myeloma. We offer the results of a retrospective analysis of MM patients with autologous transplantation in our center in the last decade. The median age was 55 years and there was male dominance in our study. The median age was relatively young in our study, similar to the study of O’Shea et al. [11].

Recent population-based studies have shown increasing use of ASCT in elderly patients with MM [12]. However, different age cut-off values of 60 years, 65 years, or 70 years were given that estimate survival independently in different studies [13,14].

In our study, ASCT was planned for transplant-eligible patients who had adequate stem cell collection. We mostly applied cyclophosphamide and G-CSF as the mobilization regimen. The target CD34+ stem cell dose for collection was >2x10^6^ CD34/kg for each autologous transplantation. The mean CD34+ cell number was (7.5±5.3)x10^6^/kg in our study. The mean platelet engraftment days were 13.9 and the mean neutrophil engraftment days were 11.5 in our study. In other studies, the median time to neutrophil engraftment and the median time to platelet engraftment were reported as 9-14 days [15] and 13.5-25 days [16], respectively. The prior studies used the total infused CD34+ cells as a predictor of neutrophil and platelet engraftment. We also demonstrated a significant correlation between the infused CD34+ cell dose and the period to platelet engraftment and neutrophil engraftment [17,18,19].

The collected stem cell product is mostly given as a single day infusion, but in some situations, the product can be reinfused on multiple days due to patient characteristics or concern about complications related to higher volumes.

We chose multi-day reinfusion to overcome possible volume overload in older patients. DMSO toxicity could be another problem if the patient is given the infusion in a single day. Patients requiring multiple days to collect an adequate number of CD34+ cells may be at risk of exposure to serious doses of DMSO. Davis et al. [20] suggested that toxicities related to the infusion of cryopreserved cells are related to the volumes of cryoprotectants, but our study did not demonstrate a difference in toxicity with multiple day infusions, like the study of Abdel-Razeq et al. [21]. We did not see any other toxicity, such as cardiac arrhythmia or volume overload complications. We also wondered about the effect of multi-day infusion on engraftment. The effect of multi-day infusion of stem cells on engraftment was evaluated in the study of Abdel-Razeq et al. [21], as well. They showed there was no effect on engraftment. However, that study consisted of a heterogeneous group of patients with non-Hodgkin lymphoma, Hodgkin lymphoma, and breast cancer, and it did not include myeloma patients. There is no other study evaluating the effect of multi-day stem cell infusion on engraftment in the literature. If we consider all patients in our study, there were no statistical differences between the two groups (multiple reinfusion days and single reinfusion day) regarding platelet and neutrophil engraftment days and hospitalization days.

We also observed that multi-day infusion of stem cells due to higher volumes was mostly done in patients older than 60 years. The ages of these patients were between 26 and 70 years; 39% of them were older than 60 years and 61% were younger than 60. On the other hand, the number of patients older than 65 years old was found to be 14%. In the Italian Group for Bone Marrow Transplantation-Working Group study, age over 65 was described as a poor mobilizing factor, like previous cytotoxic chemotherapy, radiotherapy, bone marrow involvement, and platelet count before mobilization [22]. We did not observe any problem in the mobilization of myeloma patients regarding age.

In our study, we also compared findings between two age groups (<60 years old, ≥60 years old). There were no significant differences in platelet engraftment days, neutrophil engraftment days, reinfusion days, hospitalization days, or infused CD34+ doses in these two groups. However, Sanchez et al. [23] found a significant difference in mean hospitalization days (18.6 days in older versus 16.8 days in younger patients, p<0.01) and no significant difference in hospital mortality between older and younger patients.

## Conclusion

In cases with high volumes of stem cell product to acquire adequate amounts of stem cells, reinfusion can be safely applied across multiple days without any delay in engraftment.

## Figures and Tables

**Table 1:**
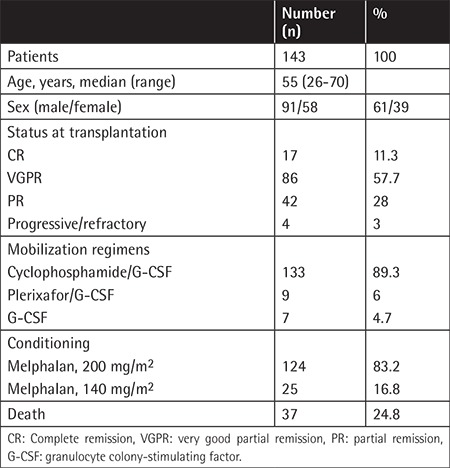
Patients’ characteristics.

**Table 2:**
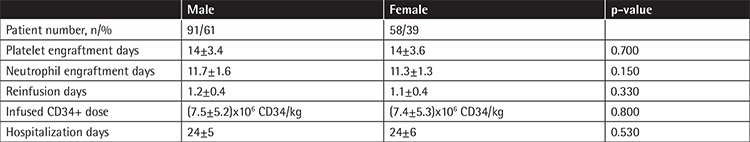
Results in regards to sex distribution.

**Table 3:**
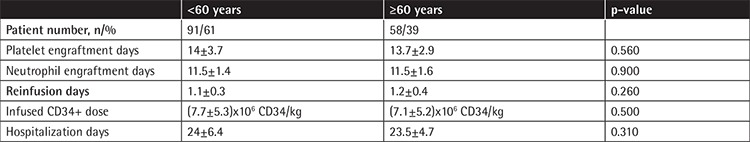
Results in regards to age (<60 years and ≥60 years).

**Table 4:**
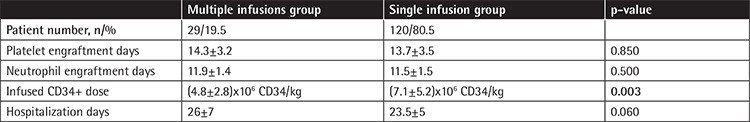
Neutrophil and platelet engraftment days, hospitalization days, and infused CD34+ cell dose according to reinfusion days.
